# Nanostructures: a platform for brain repair and augmentation

**DOI:** 10.3389/fnsys.2014.00091

**Published:** 2014-06-20

**Authors:** Ruxandra Vidu, Masoud Rahman, Morteza Mahmoudi, Marius Enachescu, Teodor D. Poteca, Ioan Opris

**Affiliations:** ^1^Department of Chemical Engineering and Materials Science, University of California DavisDavis, CA, USA; ^2^Department of Nanotechnology and Nanotechnology Research Center, Faculty of Pharmacy, Tehran University of Medical SciencesTehran, Iran; ^3^Center for Surface Science and Nanotechnology, University “Politehnica” BucharestBucharest, Romania; ^4^Academy of Romanian ScientistsBucharest, Romania; ^5^Carol Davila University of Medicine and PharmacyBucharest, Romania; ^6^Wake Forest University Health SciencesWinston-Salem, NC, USA

**Keywords:** nanotechnology, brain repair and augmentation, brain activity mapping, blood brain barrier, carbon nanotube, multi-electrode array, nano-imprint lithography, inter-laminar microcircuit

## Abstract

Nanoscale structures have been at the core of research efforts dealing with integration of nanotechnology into novel electronic devices for the last decade. Because the size of nanomaterials is of the same order of magnitude as biomolecules, these materials are valuable tools for nanoscale manipulation in a broad range of neurobiological systems. For instance, the unique electrical and optical properties of nanowires, nanotubes, and nanocables with vertical orientation, assembled in nanoscale arrays, have been used in many device applications such as sensors that hold the potential to augment brain functions. However, the challenge in creating nanowires/nanotubes or nanocables array-based sensors lies in making individual electrical connections fitting both the features of the brain and of the nanostructures. This review discusses two of the most important applications of nanostructures in neuroscience. First, the current approaches to create nanowires and nanocable structures are reviewed to critically evaluate their potential for developing unique nanostructure based sensors to improve recording and device performance to reduce noise and the detrimental effect of the interface on the tissue. Second, the implementation of nanomaterials in neurobiological and medical applications will be considered from the brain augmentation perspective. Novel applications for diagnosis and treatment of brain diseases such as multiple sclerosis, meningitis, stroke, epilepsy, Alzheimer's disease, schizophrenia, and autism will be considered. Because the blood brain barrier (BBB) has a defensive mechanism in preventing nanomaterials arrival to the brain, various strategies to help them to pass through the BBB will be discussed. Finally, the implementation of nanomaterials in neurobiological applications is addressed from the brain repair/augmentation perspective. These nanostructures at the interface between nanotechnology and neuroscience will play a pivotal role not only in addressing the multitude of brain disorders but also to repair or augment brain functions.

## Introduction

NeuroNanoTechnology (NNT) is an emerging approach in science and engineering not only to assess the unique properties, structures and functions of brain circuits, but also to manipulate or to heal damaged neural circuits. This is largely because the brain functions operate at the nanoscale level, and therefore, in order to access and communicate with the entities of interest, we need tools and techniques that work at nano-scale level as well. The synthesis and characterization, as well as the design of materials with functional organization at nanoscale give us the possibility to engineer and control functional bio-integrated systems. Importantly, the ability to manipulate atoms and molecules, induce unique properties, increase stability, and communicate signals is opening up incredible opportunities for a broad spectrum of scientists. The purpose of this review is to highlight recent engineering advances in the rapidly developing field and its clinical applications, including augmenting brain function. NNT as applied nanotechnology to engineered sensing platforms has the ultimate goal of developing interdisciplinary nanotechnology strategies that can directly investigate specific neural interactions and circuits for treating the broad spectrum of neurological and psychiatric disorders.

The foundation of the “nanoworld” was established in 1980's when for the first time, scientists were able to see the atom (i.e., the tiny “brick” of matter) in 3D real space. This was primarily due to the invention of the scanning tunneling microscope (Binning et al., 1982; Binnig et al., 1983), followed by additional techniques, such as atomic force microscopy (Binnig et al., 1986). The “nanoworld” in science consists of several nano “chapters” such as nanomaterials—materials at nanoscale; nanoarrays—arrays of nanowires; nanotools—tools needed to characterize the nanomaterials; and nanodevices—new devices, many of them using quantum effects. The miniaturization trend and the high output of integrated circuits have stimulated the development of both nanostructured materials and new synthesis methods. Thus, nano-tools bring at the table the “internal” or “external” “nano-surgeons” for operating at the nano- and micro-level in neuronal circuits. The most promising “nano-surgeons” are the carbon nanotubes (CNTs) and nanowires (NWs). Carbon nanotubes and nanowires demonstrate new and/or enhanced functions crucial to neuroscience, offering a bottom-up approach in assembling nanoscale arrays and devices.

Research efforts are concentrated towards increasing the number and the density of extracellular electrodes while decreasing the device size. Acting as “on-site” laboratories, nanostructures arrays can be integrated into sensing, stimulating, monitoring and recording devices for nano-neuroscience. For example, microelectrode arrays use nanomaterials produced for various applications including *in vivo* penetration for recording, neurostimulation and optogenetic manipulations, surface electrodes measuring event related potentials in human brain, as well as fluidic, *in vitro* chemical sensing. Although these microelectrodes are made of platinum and iridium oxide, electrochemical degradation and delamination of the coating layer of the electrode may occur in time, recent advances are resolving these problems. Neural probes and micro-devices are currently used for recording activity of large neuronal assemblies (Wise, 2007; Chang-Hsiao et al., 2010; Amaral et al., 2013). For instance, low noise 64-channel neural probes made of silicon with nanoscale leads have been demonstrated feasible by Du et al. (2011). The creation of the nanomaterials—carbon nanotubes in particular—and a general approach to the preparation and applications of nanomaterials using template synthesis are also presented in this article.

**Figure 1:**
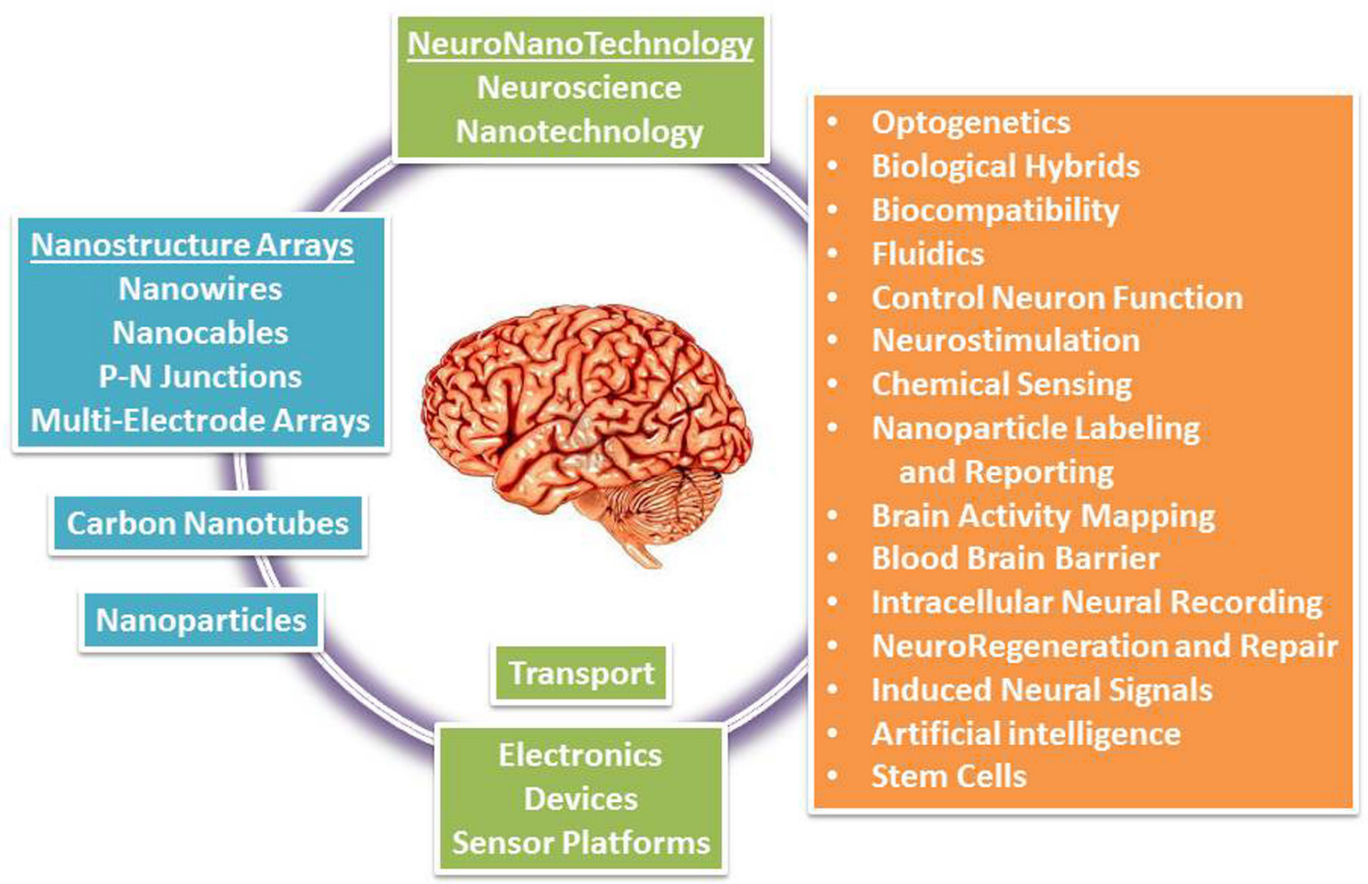
**Illustration of nanotechnology integration into the brain research**.

Additionally, recent developments in the application of nanotechnological neuroscience to the study of human brain are reviewed (Figure 1). NNT research aims to regenerate and protect the central nervous system (CNS) by developing nanoengineered substrates, for example, to help guide axon growth after damage or degeneration. Other therapeutic strategies for CNS disorders require getting a device or drug to a specific site in the CNS. Acute compression in spinal cord injury, for example, requires laminectomy and *in vivo* delivery of peptide amphiphile molecules for nanofiber network formation (in rat models) (Silva, 2005). Nanomaterials used as vessels to deliver drugs are discussed in conjunction with methods that help nanoparticles to transfer across the blood brain barrier. Finally, we review exciting advances in various clinical applications to stimulate nerve cells for regeneration and even augmentation of brain function. NNT's promise is to provide chips that will interface with the brain and allowing to detect and correcting online any potential miss-function of the brain's microcircuits bridging the perception with the executive control of behavior.

## Nanomaterials

### Carbon nanotubes

#### Nanotechnology of carbon nanotubes and nanowires junctions

Nanotubes and nanowires are nanomaterials that basically represent the quasi-one-dimensional (1D) conductors and semiconductors available for nanotechnology to use. The nanotechnology rush, currently in progress, was generated by nanotubes and nanowires that have evolved into some of the most intensively studied materials (Mao et al., 2013). Carbon nanotubes (CNTs), discovered by Iijima (1991), are exhibiting outstanding mechanical, thermal, and conductive properties. Rolling-up one or more graphene sheets generates CNTs with excellent chemical and thermal stability, extreme electronic properties, large surface area and high mechanical strength while carrying ultralight weight (Ajayan, 1999).

**Figure 2:**
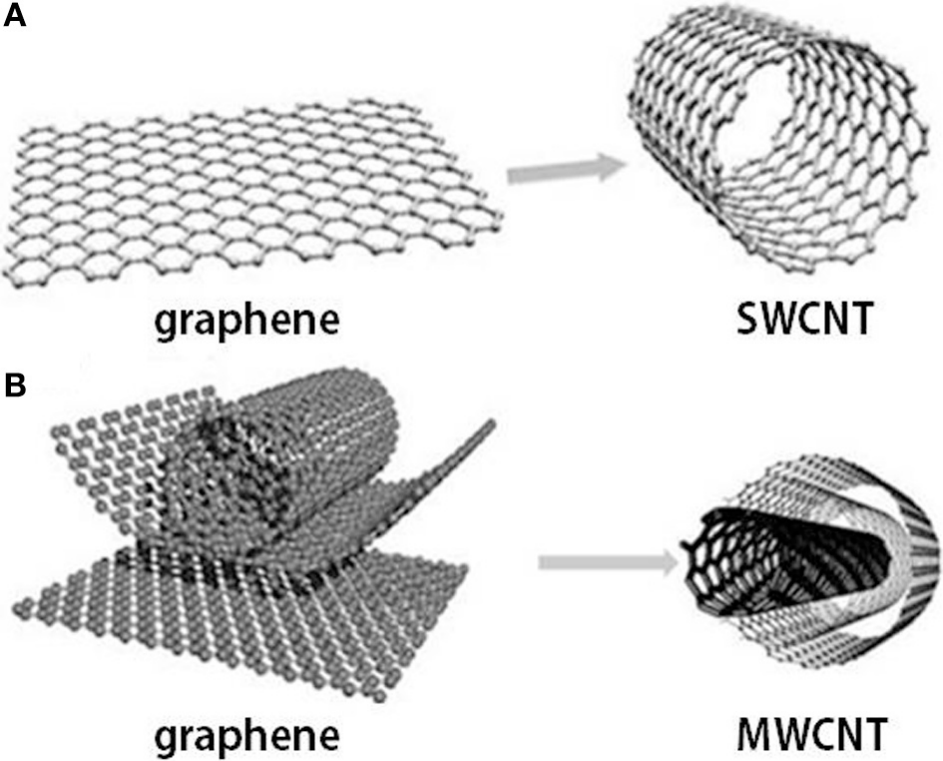
**Graphene and carbon nanotubes as (A) single wall carbon nanotube (SWCNT) and (B) multi-wall carbon nanotube (MWCNT) structures**.

Under well-defined conditions of synthesis, two forms of CNTs can efficiently be prepared: single-wall carbon nanotubes (SWCNTs) or nested multiwall carbon nanotubes (MWCNTs) (Figure 2). Being so close to graphene, CNTs are usually near to atomic-scale perfection making CNTs chemically inert (Enachescu et al., 1999; Bota et al., 2014a,b). Although the CNTs have 1/6th of the weight of steel, similar to graphene under tension, nanotubes are two orders of magnitude stronger than steel. Computer simulations estimation of melting point of nanotubes of about 3700°C is higher than that of any metal, but close to that of graphite. SWCNTs can act as very good conductors of electrons or can show semiconducting behavior, depending on their diameter and the atomic structure of nanotubes. Even the very high thermal conductivity of isotopically pure diamond is expected to be exceeded by that of CNTs (CNTs being excellent conductors of heat) that can be perfectly positioned in the devices to dissipate heat from PC chips. Additionally, CNTs are biocompatible in many environments, similar to the related graphite.

Different technological fields are witnessing great developments in CNTs devices based on their unique properties: probes, conductive composites, nanometer sized semiconductor devices, field emission displays and radiation sources, hydrogen storage media, sensors, energy storage, and energy conversion devices (Sharma and Ahuja, 2008). Functionalization of the CNTs surface was performed during the last decade using many different approaches, some of which focused on increasing CNTs solubility and lowering their toxic effects in order to fit biomedical applications (Bianco et al., 2011), because CNTs were showing poor solubility and apparently high toxicity (Fabbro et al., 2012). CNTs have been proposed either by themselves or as components for biosensors (Wenrong et al., 2010), ion channel blockers (Park et al., 2003), biocatalysts (Feng and Ji, 2011), photo-thermal probes in cancer therapy (Moon et al., 2009), nanovectors (Klumpp et al., 2006) and imaging applications (Kam et al., 2005a; Wu et al., 2005; Klumpp et al., 2006).

A nanotube can be conjugated with multi-functional agents (Wu et al., 2005). The particularity of CNTs to have a higher surface area to volume ratio compared to spheres allows high loads of therapeutic agents (Kam et al., 2005a). CNTs are very good candidates for new delivery vehicles as well increasing the therapeutic effect of drugs. Viral vectors, lipids (positive charged), polymers, liposomes, and NPs represent the previous delivery vehicles. However, reduced penetration into the cell of certain therapeutic agents is one issue of concern despite the versatility of shape, size and materials of non-viral vehicles (Endo et al., 1990). After surface modification via functionalization, CNTs show low cytotoxicity as measured over a few days (Kam et al., 2004; Lu et al., 2004; Pantarotto et al., 2004a,b; Cai et al., 2005; Kam and Dai, 2005; Kam et al., 2005a; Wu et al., 2005) while CNTs are readily internalized by cells.

In addition, single-walled carbon nanotubes (SWCNTs) show confined heating at near infrared (NIR) absorption because SWCNTs absorb strongly in NIR wavelengths range (Endo et al., 1990). In his work, Kam et al. (2005b) showed that SWCNTs released DNA upon exposure to NIR radiation, which permits its translocation into the cell nucleus. Cell death was demonstrated in the same study by using the same technique. SWCNTs were adopted by folate labeled cells, increasing the CNTs functionalization using a folate moiety.

The transport of nanotubes into cells is of fundamental importance for the biomedical applications mentioned above and below. As yet, the way in which CNTs enter cells is still under hot debate, generating both controversy and confusion about the mechanism of entering cells. Bianco et al. (Pantarotto et al., 2004b) suggested that ammonium-functionalized SWCNTs and MWCNTs are formed via a passive, endocytosis-independent mechanism; however, Dai et al. (Kam et al., 2004) came to the conclusion that the mechanism of the acid-functionalized SWCNTs entering the cell involves an endocytosis pathway. Another mechanism proposed for MWCNTs, which cannot use the endocytosis pathway due to their size takes into consideration the flipping of lipid molecules of the membrane to allow CNTs to enter the cell (Kam et al., 2004; Lopez et al., 2004; Lu et al., 2004; Pantarotto et al., 2004a,b; Cai et al., 2005; Kam and Dai, 2005). The communication between cell and nanotube is constrained by the type of coating on the nanotube surface. By transferring CNTs into cells, proteins are absorbed to the nanotube surface, coating the nanotube with serum-containing proteins, such as albumin and fibronectin. It has been suggested that the CNT transfer into cells has a natural switching mechanism of lipids in the membrane (Lopez et al., 2004; Pantarotto et al., 2004a) and to not exceed an endocytotic pathway for the MWCNTs that are 200 nm in length with 10 nm radius.

An open subject remains regarding what happens to CNTs once they have entered the cell and also about when or how they would be exocytosed by the cells (Sakhtianchi et al., 2013). The possibility for the nanotubes to be subsequently expelled from the cell would be advantageous for most biological applications; however, as yet, this has not been reported in the literature. There is still much work necessary to understand the CNTs cellular transport in order to be able to control the CNTs placement inside cells.

#### Nanowires junctions

In biology the range of length scale varies by orders of magnitudes—from nanometer sized nucleic or amino acids to several centimeters for organs and neuronal circuits. There is a need for interfaces with nanoscale spatial resolution in order to investigate processes at the subcellular level. Besides carbon nanotubes, these interfaces can be achieved through the use of other nanostructures, such as semiconductor nanowires (NWs). With dimensions that are as small as a protein molecule, CNTs and NWs present the building blocks for nanoscale electronics (McEuen et al., 2002; Lieber, 2003). The critical feature sizes (atomic scale) of these building blocks can be well-controlled during synthesis, in contrast with nanostructures fabricated by “top-down” process. Even for isolated CNTs transistors that have shown exceptional properties (Javey et al., 2003), large scale integration challenges remain due to difficulties in preparing pure semiconductor nanotubes. The issues faced by CNTs could be overcome by nanowires because of the reproducible control over size and electronic properties that current growth methods enable (Cui and Lieber, 2001; Cui et al., 2001a, 2003; Wu et al., 2004). A wide class of NWs have been developed, ranging from NWs based on classic semiconductors, such as silicon NWs (Chen et al., 2006a; Goncher et al., 2006; Yajie et al., 2008), GaP (Dujavova-Laurencikova et al., 2013), GaN (Lee et al., 2007), CdS and ZnS (Barrelet et al., 2003), heterostructures as Ge-Si (Xiang et al., 2006a,b), InAs-InP (Jiang et al., 2007), oxide nanowires MgO (Yin et al., 2002), Cu_2_O (Jiang et al., 2002), SiO_2_ (Yu et al., 1998; Liu et al., 2001; Zheng et al., 2002), Ga_2_O_3_ (Wu et al., 2000; Sharma and Sunkara, 2002), Al_2_O_3_ (Valcarcel et al., 1998; Xiao et al., 2002), In_2_O_3_ (Li et al., 2003), SnO_2_ (Dai et al., 2001), MnO_2_ (Wang and Li, 2002), Sb_2_O_3_ (Guo et al., 2000), TiO_2_ (Seraji et al., 2000; Miao et al., 2002), ZnO (Tian et al., 2003; Vayssieres, 2003), and LiNiO_2_ (Zhou et al., 2002).

The field-effect transistors (FETs) configuration of semiconductor NWs is one of the most appropriate detection schemes (Cui and Lieber, 2001; Cui et al., 2001b, 2003; Zheng et al., 2004; Xiang et al., 2006a). Binding to the dielectric gate of a polar/charged species appears analogous to applying a voltage to a gate electrode. For example, accumulation of positive carriers (holes) together with an increase/variation in device conductance can be generated by binding a protein with negative charge to the surface of a *p*-type FET. Silicon based NWs (or composed of other types of semiconductors) also may function as FET devices (Cui and Lieber, 2001; Cui et al., 2003; Lieber, 2003; Zheng et al., 2004; Li et al., 2006; Xiang et al., 2006a). One-dimensional morphology of NWs is the main feature that determines overcoming sensitivity limitations for planar FET devices. Thus, a more substantial change in device conductance for the NW vs. a planar FET will take place if any analyte binding event will happen (this event leads to accumulation or depletion of carriers).

One of the most powerful and versatile platforms based on NWs devices has emerged to build functional interfaces for biological (including neurons) systems. NWs are non-invasive (highly local) probes of neuronal projections; individual NWs devices are becoming optimal for interfacing with neurons due to the fact that the contact length along the axon (or the dendrite projection crossing a NW) is just about 20 nm. Compared to other electrophysiological methods, with micro-fabricated electrodes and planar FETs, the active junction area for NWs devices is orders of magnitude smaller and is quite similar to natural synapses. This small size creates advantages, such as: (a) spatially resolved signal detection without complicated averaging of extracellular potentials that change over a large portion of a given neuron, and (b) integration of axon's elements together with the dendrite projections from a single cell. The stimulation of neuronal activity through NW/axon junctions is also achievable using NWs devices. Somatic action potential spikes detected with intracellular electrodes, are generated by applying excitatory sequences of biphasic pulses to the NWs of NW/axon junctions (Patolsky et al., 2007).

Also, NW-based FET device can be designed into a device array; neuron growth over dense NWs device arrays is usually achievable nowadays (Patolsky et al., 2007). Thus, interfacing ensembles of NWs inputs and outputs to different neural networks and neurons enables the implementation of stimulation, inhibition, or reversibly blocking signal propagation through specific pathways (while the signal flow is simultaneously mapped throughout the network). Besides single NW-based FET devices or arrays of NW-based FET devices used for investigating neuronal activity, the NWs are also used to design and build NWs-based electrodes for neural recordings in the brain.

The potential to revolutionize neuroscience research and clinical therapy (Benabid, 2007; Kipke et al., 2008; Vaadia and Birbaumer, 2009; Suyatin et al., 2013) is represented even by implantable neural interfaces (Rutten, 2002; Fromherz, 2003; Cogan, 2008). However, the recorded neurons and tissue reactions that encapsulate and insulate the implant are still presenting instability results (Schouenborg, 2011). The nanostructured electrodes are considered as a promising alternative to conventional neuronal interfaces because the recording properties depend, primarily on electrode surface properties and tissue reactions to the surface (Kotov et al., 2009; Timko et al., 2010; Dvir et al., 2011; Voge and Stegemann, 2011; Suyatin et al., 2013). Nanostructured electrodes provide additional advantages such as improved electrical properties (Keefer et al., 2008; Cellot et al., 2009; Martin et al., 2010; Ansaldo et al., 2011; Duan et al., 2012), shorter cell-to-electrode distance (Tian et al., 2010; Duan et al., 2012; Xie et al., 2012), as well as a better spatial resolution. They also have a potential for less tissue damage (Almquist and Melosh, 2010; Martin et al., 2010; Tian et al., 2010; Duan et al., 2012), better biocompatibility (Hallstrom et al., 2007; Kim et al., 2007; Martin et al., 2010; Berthing et al., 2011) and new functionalities, such as selective guidance of neuronal fibers (Hallstrom et al., 2009). Importantly, recent cell signal recordings with different nanowire-based electrodes have been achieved *in vitro* (Tian et al., 2010; Timko et al., 2010; Brueggemann et al., 2011; Dvir et al., 2011; Duan et al., 2012; Robinson et al., 2012; Xie et al., 2012), demonstrating the epitaxially grown wires of small diameter may provide minimally invasive tissue penetration (Kawano et al., 2010; Takei et al., 2010; Tian et al., 2010; Duan et al., 2012; Xie et al., 2012). Up to now, using mainly carbon nanotubes without structural feature control and in combination with rather big surfaces has been performed with *in vivo* studies using nanostructured neuronal electrodes (Keefer et al., 2008; Ansaldo et al., 2011; Suyatin et al., 2013). However, recently, it has been shown that with neuronal interfaces for improved cell survival (Hallstrom et al., 2007) and improved cell adhesion with focal adhesions forming specifically on the nanowires, the epitaxially grown gallium phosphide (GaP) NWs have beneficial properties (Prinz et al., 2008). Compared to other material NWs, GaP NWs can be synthesized with very little tapering and exceptional control over their position and geometry, and with a high aspect ratio (over 50) (Suyatin et al., 2009). Also recently, the design and fabrication of a first generation of GaP NW-based electrode with a controllable nanomorphology was reported (Suyatin et al., 2013). The first functional testing *in vivo* of a NWs-based device was performed during acute recordings in the rat cerebral cortex, where the NWs were used as a backbone for a metal nanostructured electrode with a three-dimensional (3D) structure. This electrode design opened the development of a new model system, with the prospect of enabling more reliable tissue anchoring as well as a more intimate contact between the electrode and the neurons, Xie et al., (2010) furthering research on the functionality of nanostructure-based neuronal interfaces *in vivo*, given the better electrode-cell electrical coupling (Hai et al., 2010; Robinson et al., 2012; Xie et al., 2012).

In recent years, a broad platform for electronic interfaces with cells and tissue using CNTs and NWs devices has been implemented. Compared to standard techniques used to measure, record and observe extracellular signals from individual tissues and cells, CNTs and NWs devices have orders of magnitude smaller recording area. The mV range signals of CNT/NW platforms device are significantly larger than those measured using planar devices or multiple electrode arrays, likely due to enhanced coupling between the cell membrane and nanoscale device. CNTs and NWs represent also the natural building blocks for biological-nanomaterial interfaces; the creation of hybrid nanoelectronic-neuronal devices is permitting novel directions in neuronal research and applications. The possibility of tuning their material composition and corresponding properties at the time of synthesis is opening up the design of ultra-high sensitivity devices at nanoscale for future opportunities.

#### Carbon nanotubes use in neuroscience

Carbon nanotubes have an arsenal of properties (electrical, mechanical, and chemical) that make them very promising materials for applications in neuroscience. The ease with which carbon nanotubes can be patterned makes them very useful for studying the organization of neural networks while the electrical conductivity of nanotubes can provide a vital mechanism to monitor or stimulate neurons. Carbon nanotubes themselves can interact with and affect neuronal function, most likely at the level of ion channels (Malarkey and Parpura, 2007, 2010). Both SWCNTs and MWCNTs have been increasingly used as “scaffolds for neuronal growth.” Lately, CNTs were used in the research of neural stem cell growth and differentiation. Additionally, CNTs were applied as interface materials with neurons, where they deliver electrical stimulation to these cells and detect electrical activity. Here are just few applications of the CNTs:

***Interfacing cultured neurons with carbon nanotubes***. To demonstrate that the electrical simulation produced by single-wall carbon nanotubes (SWCNTs) can indeed induce neuronal signaling, Mazzatenta et al. (2007) developed an integrated SWCNTs neuronal system and demonstrated that hippocampal cells can be grown on pure SWCNTs substrates. Their experimental results point to the fact that SWCNTs can be directly used to stimulate brain circuit activity. These results may have a remarkable impact on the future developments and architectural design of microsystems for neural prosthetics (Mazzatenta et al., 2007).

***Intracellular neural recording with pure carbon nanotubes probes***. A novel millimeter-long electrode, remarkably simple, can be used to produce extracellular and intracellular recordings from vertebrate neurons *in vitro* and *in vivo* experiments, when it is terminated with a tip fabricated from self-entangled pure CNTs with sub-micron dimensions (Yoon et al., 2013). Assembling intracellular electrodes from CNTs using the self-entangled CNTs fabrication technology is opening a new opportunity to harness nanotechnology for neuroscience applications.

***Carbon nanotubes in neuro-regeneration and repair***. CNTs based technologies are emerging as particularly innovative tools for tissue repair and functional recovery after brain damage, due to their ability to interface neuronal circuits, synapses and membranes (Sakhtianchi et al., 2013). Carbon nanotube technology can now be applied to develop new devices that are capable to drive repair of nerve tissue, neuronal differentiation, growth and network reconstruction.

***Analog neuromorphic modules based on carbon nanotube synapses***. Shen et al. (2013) recently reported an analog neuromorphic module consisting of an integrate-and-fire circuit and *p*-type carbon nanotubes (CNTs) synapses. The CNTs synapse resembles a FET structure using as its gate an aluminum oxide dielectric layer implanted with indium ions and as its channel a random CNTs network. Electrons are attracted into the defect sites of the gate aluminum oxide layer by a positive voltage pulse applied to the gate, followed by a gradual release of the trapped electrons after the pulse is removed. Thus, the electrons induce a dynamic postsynaptic current in the CNTs channel by modifying the holes' concentration. The excitatory or inhibitory postsynaptic currents generated by the multiple input pulses via excitatory or inhibitory CNTs synapses flow toward an integrate-and-fire circuit which triggers output pulses. Further, the analysis of the dynamic transfer functions between the input and output pulses of the neuromorphic module are performed. An emulation of biological neural networks and their functions could be implemented by scaling up such a neuromorphic module.

***Nanotechnology and nanocomputing***. The last decade in nanotechnology research witnessed an increasing use of artificial intelligence tools (Sacha and Varona, 2013). Current and future perspectives in the nanocomputing hardware development can boost the field of artificial-intelligence-based applications. Moreover, convergence of the two sciences, i.e., nanocomputing and nanotechnology, has the potential to shape research directions and technological developments in medical and information sciences. The great potential of combining nanotechnology and nanocomputing is also shown by hybrid technologies (i.e., nanodevice and biological entities), neuroscience, bioengineering combined with new data representations and computer architectures and a large variety of other related disciplines.

#### Carbon nanotubes platform for regeneration, stroke, brain tumors, and neoplasm

Carbon nanotubes present a broad regenerative spectrum from nerve tissue repair to bone tissue engineering (Newman et al., 2013).

***Neuroregeneration and repair***. Development of future strategies for tissue repair in order to promote functional recovery after brain damage is one of the main aims of nanotech studies (Fabbro et al., 2012). In this framework, particularly innovative tools are emerging based on CNTs technologies due to their ability to interface with neuronal circuits, synapses and membranes, as well as due to the outstanding physical properties of these nanomaterials. CNTs technology is now applied to the development of devices able to drive nerve tissue engineering, focusing in particular on growth and nerve network reconstruction, neuronal differentiation and nerve tissue repair.

Arslantunali et al. (2014) constructed a nerve conduit from poly (2-hydroxyethyl methacrylate) (pHEMA) that was loaded with MWCNTs. This pHEMA guide was more hydrophobic and more conductive than pristine pHEMA hydrogel when loaded with relatively high concentrations of MWCNTs (6%, w/w in hydrogels). The neuroblastoma cells seeded on pure pHEMA lost their viability when an electrical potential was applied, while MWCNTs carrying pHEMA maintained their viability, demonstrating that MWCNTs are conducting electricity, making them more suitable as nerve conduits. CNTs are instrumental in regeneration and reparation of irreversibly diseased or damaged nerve tissues in the peripheral and central nervous system of the human body (Stankova et al., 2014).

A class of ideal biomaterials for a wide range of regenerative medicine applications is MWCNTs polymer composites because they are hybrid materials combining numerous electrical, mechanical and chemical properties. Using a composite as a substrate to increase electronic interfacing between neurons and micro-machined electrodes (Antoniadou et al., 2011) reported the synthesis and characterization of a novel biomaterial for the development of nerve guidance channels in order to promote nerve regeneration, opening up potential applications for prosthetic devices, neural probes, and brain implants.

***Stroke damage repair***. Disruption of tissue architecture happens as a result of a stroke. However, in a rat stroke model, amine-modified SWCNTs protect neurons from injury. CNTs used as scaffolds in brain tissues and neural cells have shown promising results, supporting the treatment strategy based on transferring stem cells containing scaffolds to damaged regions of the brain. In rats with induced stroke, protection of neurons and enhanced recovery of behavioral functions were observed for the rats pretreated with amine-modified SWCNTs (Lee et al., 2011a). Also, the amine-modified SWCNTs protected the brains of treated rats, as indicated by the low levels of apoptotic, angiogenic and inflammation markers. In another study, it was shown that CNTs promote recovery from stroke when they are impregnated with neural progenitor cells in subventricular zones. The improvement of stem cell differentiation to heal stroke damage assisted by CNTs was first demonstrated by Moon et al. (2012).

***Cancer and brain tumors therapy***. It has been shown that the intratumoral delivery of low-dose of free CpG is less effective than immunostimulatory CpG oligodeoxynucleotides conjugated with (CNT-CpG) (Adeli et al., 2012). Moreover, CpG oligodeoxynucleotides conjugated with (CNT-CpG) was shown to induce antitumor immunity that protect mice from subsequent systemic or intracranial (i.c.) tumor rechallenge as well as eradicating i.c. gliomas. Also, it was shown that the treatment of metastatic brain tumors could be more efficient using the same “intracerebral immunotherapy” strategy. Thus, compared to systemic therapy generating antitumor responses that target both brain and systemic melanomas, the intracerebral CNT-CpG immunotherapy is more effective. Moreover, CNTs potentiate CpG.

A novel type of nanoprobe employs SWCNTs as a photosensitizer for application in cancer cell imaging and therapy. Ou and Wu (2013) developed a nanoprobe based on SWCNTs and a fluorescent photosensitizer pyropheophorbide (PPa) for use in cancer cell imaging and therapy *in vitro*. Phospholipids bearing polyehylene-glycol modified SWCNTs which provide an interface for the conjugation of PPa were prepared by ultrasonication. The polyehylene-glycol modified SWCNTs were then conjugated with PPa using covalent functionalization methods to construct a SWCNTs-PEG-PPa nanoprobe. The functionalization of SWCNTs was evidenced by UV-vis absorption spectra and fluorescence spectra. Imaging cancer cells with SWCNTs-PEG-PPa nanoprobe was confirmed using two cancer cell lines via laser scanning confocal microscope tests, and killing cancer cells with SWCNTs-PEG-PPa was demonstrated using cytotoxicity tests. This indicated that the resulting SWCNTs-PEG-PPa nanoprobe has great potential to be a potent candidate for cancer imaging and therapy. Also, CNTs-polymer interactions play a key role in cancer therapy (Adeli et al., 2012).

### Nanomaterials and blood brain barrier

#### Transportation mechanisms through the brain barrier

**Figure 3:**
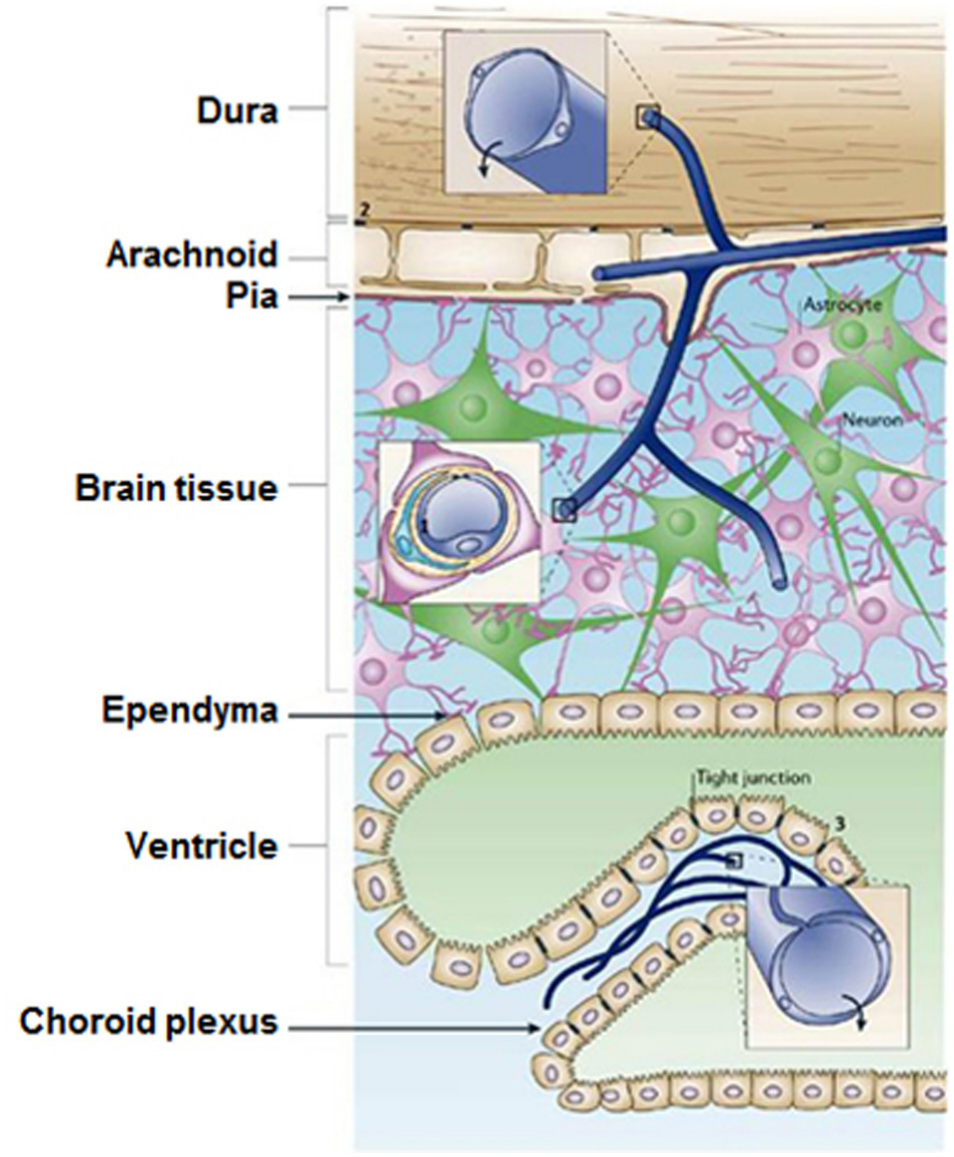
**The locations of tight junction barriers in the central nervous system. (1)** brain endothelium forming the BBB, **(2)** the arachnoid epithelium forming the middle layer of the meninges, and **(3)** the choroid plexus epithelium which secretes cerebrospinal fluid. (Reprinted by permission from Macmillan Publishers Ltd: Abbott et al., 2006.)

One of the major challenges for nanotechnology deals with the diagnosis and treatment of BBB-related dysfunctions involving stroke, brain tumors and cancer. Tight junction (TJ) barriers protect the CNS. These barriers are located in three main locations inside CNS: the brain endothelium, the arachnoid epithelium, and the choroid plexus epithelium (Figure 3, Abbott et al., 2006). BBB consists of endothelial cells connected by close fitting junctions that separate the flowing blood from the brain extracellular fluid. Therefore, BBB controls the entrance of biomolecules into the brain and protects the brain from many common bacterial infections. However, the BBB presents a few limitations for nanomedicine approaches. For instance, due to the presence of BBB, the drug delivery to the brain area for tumor therapy or other neurodegenerative diseases such as Alzheimer's is a crucial challenge. The majority of diagnosed brain tumors are currently treated with surgery, radiation, and chemotherapy; nanoscience and technology could be a promising solution to this challenge. There are several comprehensive reviews on the interaction of BBB with nanomaterials that focus on various methods to transfer nanomaterials across BBB (Chen and Liu, 2012; Khawli and Prabhu, 2013; Krol et al., 2013).

**Figure 4:**
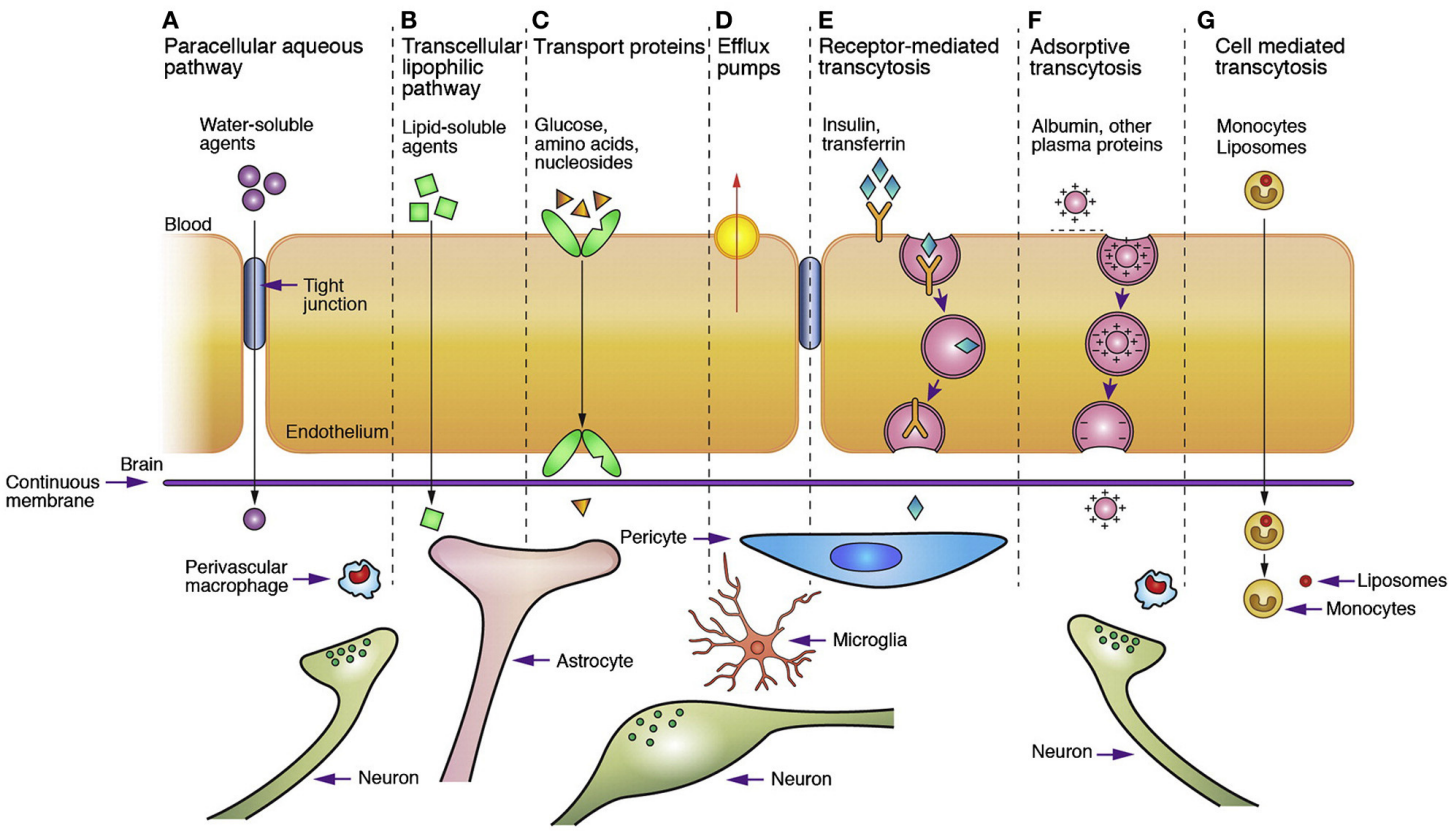
**Transport pathways across blood brain barrier**. (Reprinted by permission from Macmillan Publishers Ltd: Chen and Liu, 2012.)

Figure 4 (Chen and Liu, 2012) presents the main, well-recognized, transport pathways across BBB, which are commonly used for carrying solute molecules. Among all the pathways shown in Figure 4, the “g” route is the most suitable for drug delivery via liposomes or nanoparticles. A summary of the conventional methods used for BBB permeability assessment is given in Stam's work (Stam, 2010).

Different approaches and routes possible for transport of drugs across the BBB as summarized in Table 1. Biocompatible nanomaterials such as nanoparticles, liposomes, and supramolecular aggregates are promising drug carriers since their size can be tuned to fit the BBB transport. In addition, their surfaces can be functionalized to facilitate their transport through the BBB. It should be mentioned that the cytotoxicity of NPs must be precisely monitored, using various well-recognized methodologies (Mahmoudi et al., 2010, 2011a; Mao et al., 2013), to ensure their biocompatibility. The surface functional groups enhance the BBB permeability by various mechanisms such as adsorptive-mediated transcytosis and receptor-mediated transcytosis. As an example, Lactoferrin is a receptor located on cerebral endothelial cells that facilitates the transport of NPs across BBB by receptor-mediated transcytosis (Qiao et al., 2012).

**Table 1:** **Possible methods and routes for drug transport across BBB**.

**Method**	**Examples of studied drug/marker**	**Remarks**	**References**
Ultrasound-assisted TJ opening	Evans Blue Dye transport through BBB of white rabbits fluorescent-tagged dextrans at different molecular weights in mice	Transient, localized, reversible disrupt of BBB by ultrasonic High-frequency focused ultrasound results in skull overheating and skull-induced beam distortion low-frequency ultrasounds may produce standing waves inside the human skull, which might result in intra-cerebral hemorrhage low-frequency ultrasound require longer exposure time (5) Drug can be loaded inside micro-bubbles	Choi et al., 2010; Liu et al., 2010; O'Reilly and Hynynen, 2012; Ting et al., 2012; Beccaria et al., 2013; Burgess and Hynynen, 2013
Electromagnetic field-assisted TJ opening	Markers such as Fluorescein, Albumin, Mannitol, Evans Blue, Sucrose, horseradish peroxidase	Pulse wave is more effective than continuous wave in BBB permeability Macromolecule permeability can be reversibly increased by high electromagnetic fields (EMF), which also increase by more than 1°C the brain temperature Data on low frequency EMF (without tissue heating) is sparse and does not depend on permeability changes EMF could induce overexpression of beta amyloid	Qiu et al., 2010; Stam, 2010; Jiang et al., 2013
Macrophage-assisted TJ opening	HIV-1 encephalitis rodent model macrophage bearing liposomal doxorubicin	Monocytes/macrophages can reach the tumor sites across BBB by acting as Trojan horses carrying drug cargoes	Dou et al., 2009; Choi et al., 2012
Protein-assisted TJ opening	Fluorescein isothiocyanate-dextran	Twin-arginine translocation (Tat) translocase proteins TatB and TatC disrupt the BBB integrity	Gandhi et al., 2010
Peptide-assisted TJ opening	Small interfering RNA for gene silencing	There is a 29-amino-acid peptide that can be bound specifically to the acetylcholine receptor, i.e., neuronal cells	Kumar et al., 2007
Surfactant-assisted TJ opening	Digoxin	Pluronic block copolymer P85 inhibited the drug efflux from brain via P-glycoprotein efflux mechanism	Batrakova et al., 2001
Functionalized Nanocarriers	Nanomaterials as drug carriers has been reviewed in several papers. The requirements for this application are: (1) Stable in blood and long blood circulation time, (2) tunable drug release, (3) BBB-targeting mechanism		Tysseling and Kessler, 2011; Chen and Liu, 2012; Wohlfart et al., 2012
Amphiphilicsupramolecular aggregates	Beta-galactosidase as a model protein	Vesicles, micelles, and liposomes are frequently used in drug delivery Pluronic has also been used as nanocarrier which upon conjugation with Chitosan is effective for delivery of proteins to the brain Polyethylene glycol increase the life time of liposome by preventing interaction/exchange with cell membranes as well as protection against Phagocytes	Kumari et al., 2010; Kim et al., 2013; Kreuter, 2013
Transport vectors	L-DOPA	The route for transport of nutrients to brain can be used as successful strategy. But this method is limited to peptide drugs with similar molecular structure to nutrients	Wade and Katzman, 1975
Adsorptive-mediated transcytosis (AMT)	siRNA	Cell penetrating peptide and cationic proteins use AMT to enter the brain	Adenot et al., 2007; Sharma et al., 2012; Kanazawa et al., 2013
Endogenous receptor- mediated transcytosis (RMT)	RMT has the advantage of BBB targeting. The targeting starts with endocytosis after receptor-ligand binding which is followed by exocytosis to the brain side. Different receptors employed are: (1) Insulin receptor, (2) Transferrin receptor, (3) Lipoprotein receptors, (4) Diphtheria toxin receptor		Qiao et al., 2012; Wang et al., 2013; Wiley et al., 2013
Cell-mediated transport	Cells such as macrophages and monocytes act like Trojan horse to transport the drug		Jain et al., 2003

In addition to the brain tumors which require drug delivery to the brain, there are some diseases which are related to dysfunctional BBB (Azhdarzadeh et al., 2013). The BBB diseases are epilepsy, Meningitis, Alzheimer's disease, multiple sclerosis (MS), brain abscess, Neuromyelitisoptica, Progressive Multifocal Leukoencephalopathy (PML), late-stage neurological trypanosomiasis (Sleeping sickness) and *De Vivo* disease (Mahmoudi et al., 2011b, 2012a; Amiri et al., 2013a). Nanomaterials show promising results for treatment of these diseases. Some of the recent nanomaterials investigations in BBB-related disease are summarized in Table 2.

**Table 2:** **Some of the recent investigations of NPs in the treatment of BBB-related diseases**.

**Type of NP/Disease**	**Physicochemical properties**	**Results**	**Remarks**	**References**
Cationic antimicrobial peptide/meningitis	CG3R6TAT, the short amphiphilic peptide forms micelles with TAT molecules toward the external medium, having the hydrophilic peptide shell and hydrophobic cholesterol core	NPs crossed the BBB and inhibited the evolution of bacteria in the infected parts of brain, a high therapeutic index (50) against S. aureus infection in a rabbit model was noticed. NPs also showed the same efficiency in decreasing the growth rate of C. neoformans in brain compared to amphotericin B as they do not damage the kidney and liver and do not change the blood electrolyte balance	TAT peptides (YGRKKRRQRRR) help the NP to cross the BBB	Liu et al., 2009; Wang et al., 2010
Amphotericin B-polybutylcyanoacrylate NPs (AmB-PBCA-NPs)/cryptococcal meningitis	NPs with mean particle diameter of 69 nm were modified with polysorbate 80	The NPs path through BBB was perceived after 30 min. Mice treated by AmB-PBCA-NPs lived more than 4 days. Up to 80% survived to the day 10 remaining constant until day 20th		Xu et al., 2011
Anti-body conjugated iron oxide NPs/ multiple sclerosis	Iron oxide macroparticles of around 1 μm conjugated with rat antibody of vascular cell adhesion molecule-1 (VACAM-1)	Enhanced detection resolution of VACAM-1 at early stage	Early stage MRI molecular imaging of disease activity with contrast agent	McAteer et al., 2007
Melarsoprolnanosuspension/cerebral stage of African trypanosomiasis	Sizes of 324 and 427 nm	Decreasing the concentration of melarsoprol in brain but increasing the concentration at bone marrow, the nanosuspension decreases the brain toxicity, which may not be useful for Trypanosomiasis; nevertheless, it might be helpful for Leukemia treatment	*In vitro* data also showed that nanosuspension concentration in the bone is very much higher	Ben Zirar et al., 2008
Porous cationic NP having oily core (70DGNP+)/ Trypanosomabrucei. African trypanosomiasis	NPs with zeta potential of 29 mV and an average diameter of 74 nm	*In vitro* tests proved efficacy	Loading the diminazene inside NPs after the synthesis of NPs resulted in better entrapment with a stability up to 6 months and about 80% entrapment efficiency	Kroubi et al., 2010
Lipid–diminazene conjugate NPs/Trypanosomiasis	Multiple particle sizes within the range of 285–442 nm and zeta potential around −35 mV	The adsorption pattern of plasma protein indicates a higher uptake chance of the receptors at BBB		Olbrich et al., 2004
Ag-nanotriangles on the surface of functionalized mica/Alzheimer's Disease	Ag NPs with width and length of 90 and 25 nm, respectively	Quantitative detection of amyloid-β at physiologically monomer concentration	Use of localized surface plasmon resonance for optical biosensor	Haes et al., 2005
Amine-modified single-walled carbon nanotube (a-SWCNT)/Stroke	a-SWCNT prepared from SWCNT (4–10 nm in diameter and 500–1500 nm in length)	There is a low damage of the tissue in treated rats. SWCNT can protect from ischemic injury due to the low levels of apoptotic, antigenic and inflammation markers	Carbon nanotubes as scaffold of neural cells	Lee et al., 2011b

#### Hidden factors

Several “ignored” factors exist at the nano-bio-interface such as the effects of protein corona, cell “vision,” gradient plasma concentration, and temperature (Laurent et al., 2012; Mahmoudi et al., 2012b; Amiri et al., 2013b; Ghavami et al., 2013). In order to have high-yield NP delivery to the brain environment, these crucial hidden factors should be carefully considered. The protein corona is a tightly formed layer of proteins at the surface of nanomaterials at their entrance into the biological fluids (such as blood plasma) (Monopoli et al., 2012). Thus, the biological species (e.g., cells) interact with the corona-coated NPs, rather than the pristine surface-coated one. In this case, *in vitro* models evaluating NPs for brain-related diseases should use the corona-coated NPs to reflect the real *in vivo* situation (Mahmoudi et al., 2012c, 2013a,b, 2014). As mentioned earlier, engineering the surface of drug carriers with functional groups to enhance BBB targeting and transport is one of the main approaches to reach therapeutic agents to the brain environment (as confirmed by *in vitro* BBB models) (Ragnaill et al., 2011). However, the protein corona may cover the designed functional groups and significantly reduce the ability of NPs to cross through cell barriers (Laurent and Mahmoudi, 2011; Mirshafiee et al., 2013; Salvati et al., 2013). Thus, in order to design NPs with high BBB cross-ability yield, one should control the corona composition *in vivo*. As the composition and the structure of protein corona depend on the chemistry and the physics of the nanostructured materials (e.g., shape, size and distribution, crystallinity, surface composition, surface functional groups, surface roughness/smoothness, and surface charges), one can tune these characteristics to have desired proteins in the corona composition. For instance, association of apolipoprotein-E in the corona composition could enhance NPs transport across BBB (Wagner et al., 2012). Another important but ignored matter is the effect of temperature on the protein corona. Slight temperature changes may cause considerable variation of protein corona composition at the surface of NPs (Ghavami et al., 2012; Amiri et al., 2013a; Mahmoudi et al., 2013c, 2014). The mean body temperature varies slightly for healthy individuals (mainly in the range of 35.8–37.2°C) but will vary in different parts of the body in different circumstances.

For instance, in the case of disease, the body temperature may have significant variations (e.g., it can reach to 41°C in the case of fever). Therefore, one can expect to have different corona composition at the surface of the injected NPs for different individuals, leading to the various therapeutic effects. In order to have high-yield therapeutic results, the body temperature of the individuals must be tightly monitored/controlled. Additionally, local temperature changes near the surface of NPs (e.g., by laser activation of plasmonic NPs) can change the composition of protein corona (Mahmoudi et al., 2014). Therefore, one can expect that the potential changes in the protein corona following hyperthermia or laser treatment of magnetic and plasmonic NPs may change *in vivo* toxicity or biodistribution in clinical applications.

#### Emerging new therapies for stroke, tumors, and cancer

By far the best therapy is to prevent damaging/degenerative effects of any kind. For example, rare earth NPs prevent retinal degeneration (Chen et al., 2006b). It is believed that in blinding diseases such as macular degeneration or retinitis pigmentosa, as well as stroke, Alzheimer's, atherosclerosis, diabetes and other disorders, these NPs could efficiently inhibit cell death. Therefore, a unique technology for multiple diseases can be created by using NPs as a novel strategy to direct therapy for various disorders. Other applications of NPs are as follows:

***Improvement in cerebrovascular dysfunction following traumatic brain injury***. Cerebrovascular dysfunction that is characterized by a decrease in cerebral blood flow (CBF) is a critical factor that worsens after traumatic brain injury (TBI). To improve cerebral dysfunction, a new class of antioxidants (nontoxic carbon particles) based on poly(ethylene glycol)-functionalized hydrophilic carbon clusters (PEG-HCCs) has been developed. This was demonstrated in a mild TBI/hypotension/resuscitation in rat when administered during a clinical relevant event: the resuscitation (Bitner et al., 2012). A concomitant normalization of superoxide and nitric oxide levels was also noticed. This is highly relevant for patient health improvement under clinical conditions requiring resuscitative care as well as in circumstances of stroke and organ transplantation.

***Primary brain tumors: diagnosis and treatment***. Since glial tumors seem to be able to create a favorable environment for the invasion of neoplastic cells into the cerebral parenchyma when they interact with the extracellular matrix via cell surface receptors, the prognosis in patients affected by primary brain tumors is still very unfavorable. The major problem for drug delivery into the brain is due to the presence of BBB as discussed above. NP systems can represent ideal devices for delivery of specific compounds to brain tumors, across the BBB. The results described by Caruso et al. (2010) shed light on the emerging novel applications of NP systems in diagnosis and treatment of primary brain tumors, and also on the NP systems as drug delivery carriers in brain tumor diagnosis and therapy.

***Applications of boron-enriched nanocomposites in cancer therapy***. Nanocomposites have stirred much attention due to their applicability in cancer therapy. In particular the isotope ^10^B, has a unique ability to absorb a slow neutron in order to initiate a nuclear reaction with release of energetic Li-particles that was not observed in the carbon analogs. The nuclear capture reaction principle has been successfully applied in radiation therapy and further used in boron neutron capture therapy (BNCT). Thus, BNCT may be applied as a promising treatment for malignant brain tumors and in other types of cancer, regardless of the limitation in neutron sources (Yinghuai and Hosmane, 2013). Recent research demonstrated that such development of “boron-based therapeutic nanomaterials” based on BNCT agents holds promise for cancer therapy.

## Nanowire synthesis and integration

### Template synthesis

Through the years, novel technologies were developed to create nanostructures with a defined set of properties for a particular application. Template synthesis is one of the technologies that emerged in the quest for better nano-electronics. Nanostructure synthesis with a template offers the possibility to grow nanostructures with complex compositions (Quach et al., 2010; Vidu et al., 2012), high aspect ratios, and integrated junctions, such as nanocable structures with integrated p-n junctions (Vidu, 2000; Vidu et al., 2007). The nanosize of these structures, and the diameter in particular, impose a series of interesting properties to this material (Piraux et al., 1999). More importantly, template synthesis offers the direct integration of nanostructures into electronic devices. Once the template is created, nanostructures can be produced by either chemical (electroless deposition), electrochemical, or physical methods. After the nanostructures are synthesized, the template can be removed to expose the nanostructure arrays.

Presently, only polycarbonate track-etch membranes (PCTE) and porous alumina membranes (AAO) have been largely used for template synthesis. The track-etch method is a well-established way to produce micro and nanoporous polymeric filtration membranes (Martin, 1994, 1996; Martin and Mitchell, 1999; Apel, 2001; Vidu et al., 2007). Track-etch polymeric membranes are obtained by bombarding a polymeric sheet of a given thickness (between 6 and 20 μm) with heavy ions to generate tracks that are then etched with acids to form pores in the tracks. The resulting membrane contains transversal pores of uniform diameter that are randomly distributed (Nkosi, 2005). On the other hand, alumina membranes are prepared by electrochemical methods, i.e., by anodization of aluminum foil in acidic solution (Despic and Parkhutik, 1989). Unlike track-etch membranes, the pores in the AAO membrane are almost parallel to the surface normal. Typical membrane thickness is up to 100 μm but more limited in pore sizes compared to PCTE (Foss et al., 1992, 1994; Pang et al., 2002, 2003a,b,c; Tian et al., 2004).

Other nanoporous materials and membrane templates include mesoporous zeolites (Miller et al., 1988; Tierney and Martin, 1989; Beck et al., 1992), nanochannel array glass (Tonucci et al., 1992), polypeptide tubules (Ye et al., 2001), surface relief grating (SRG) templates (Ye et al., 2001; Yi et al., 2002a,b,c), and other nanoporous membrane (Ozin, 1992; Schollhorn, 1996). For example, SRG patterns can be used as a template for nanowire fabrication and colloid self-assembly. Titanium nanowires have been fabricated using a spin-on process on both flat substrates of epoxy-based azobenzene functionalized polymer (AFP) templates and on one-dimensional (1D) SRG patterns (Yi et al., 2002a,b,c).

Template synthesis involves the creation of nanowires or nanotubes inside a template using various deposition techniques. In the following, the electroless and electrochemical deposition will be discussed. In particular, the electrochemical deposition permits the synthesis of nanostructures with unique properties in an integrated approach that allows us to design the sensing device and to control the architecture of the array while reducing fabrication costs.

#### Electroless deposition

Electroless deposition can be used to create nanoelectrodes in templates that generally speaking are not conductive. Electroless deposition can be used to create nanotubes or nanowires by coating the nanopore wall or by filling up the pore with the material of interest. Nanocables with radial junctions can also be produced (e.g., Au/Te, Ku et al., 2005; Vidu et al., 2006). Slow electroless plating (no mass transfer limitations) allows for a uniform metallic film, where the metal deposition occurs uniformly at the pore walls creating hollow metallic nanotubes inside the pores (Hou et al., 1998; Bergquist et al., 2001; Bercu et al., 2004; Yuan et al., 2004a,b,c).

#### Electrochemical deposition

In recent years, electrochemical nanotechnology (nanoelectrochemistry) has become a key technology due to the scale up potential and low energy consumption. Typically, a decrease in the size of an electrode causes changes in the diffusion layer from linear to spherical form. For multiscale nanostructures such as nanotubes, nanofibers, and nanocables, it is important to know which characteristic length scale, nm or μm, governs the deposition process (Lebedev et al., 2005). For the μm scale, diffusion limitations can be important if the surface deposition processes are relatively fast.

**Figure 5:**
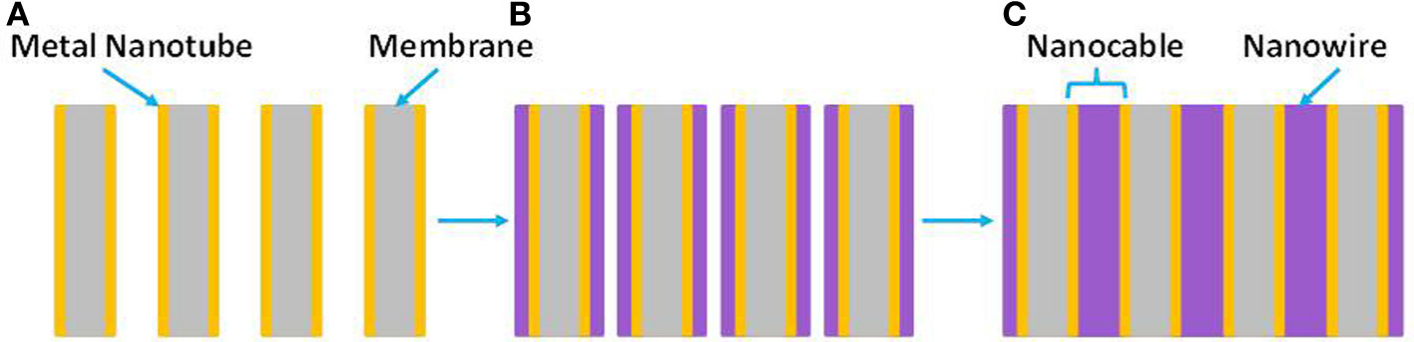
**(A)** Metal-nanotube membrane formed by electroless deposition of metal nanotubes inside the PCTE membrane. **(B)** Electrochemical deposition radially fills in the nanopores (the arrows show growth direction). **(C)** Nanocables obtained inside nanoporous PCTE membranes.

Electrochemical template synthesis is mainly used to create arrays of nanoelectrodes (Wirtz et al., 2002a,b; Wirtz and Martin, 2003; Ku et al., 2004; Quach et al., 2010; Vidu et al., 2012). Both axial and longitudinal growth of nanocables with p-n junctions can be produced. Figure 5 illustrates the process of creating nanocable structures using a combination of electroless and electrochemical deposition. There are several advantages associated with nanoelectrode arrays, which usually display a small potential drop. This behavior makes possible electrochemical measurements at low electrolyte concentrations. The small size of the nanoelectrodes array maintains a steady-state current and has a high ratio of signal to noise. This property is mainly used in sensors, where the sensitivity of the device could increase more than 100 times. Furthermore, one of the many advantages for preparing multilayered nano-sized materials is that the electrodeposition can be performed at room temperature, which is very important for systems in which undesirable interdiffusion occurs between the adjacent layers.

Electrochemical deposition can also be used to synthesize conductive polymer nanotubes and nanowires, such as polypyrrole, polyaniline, or poly(3-methylthiophene). In this case, the pore walls are favorable sites for nucleation and growth, resulting in polymeric tubules. Various polymeric structures such as thin-walled tubules, thick-walled tubules or solid fibrils can be obtained by simply controlling the polymerization time (Brendel et al., 1997; Cepak et al., 1997; Demoustier-Champagne et al., 1998, 1999; Demoustier-Champagne and Legras, 1998; Demoustier-Champagne and Stavaux, 1999).

However, more recent developments have suggested that a more sophisticated architecture of the NCs arrangement cannot be achieved using commercial templates. More complex NCs or NWs configurations for certain applications in neuroscience can use other technologies to create particular nanostructured arrays for nanoelectrodes, such as nano-imprinting. This is particularly important in designing new nanoelectronics for applications in neuroscience.

### Nano-imprinting technology

The ability to replicate patterns is of crucial importance to the advancement of micro- and nano-devices, and to induce the development of stem cells into the desired cell types (Mahmoudi et al., 2013b).

**Figure 6:**
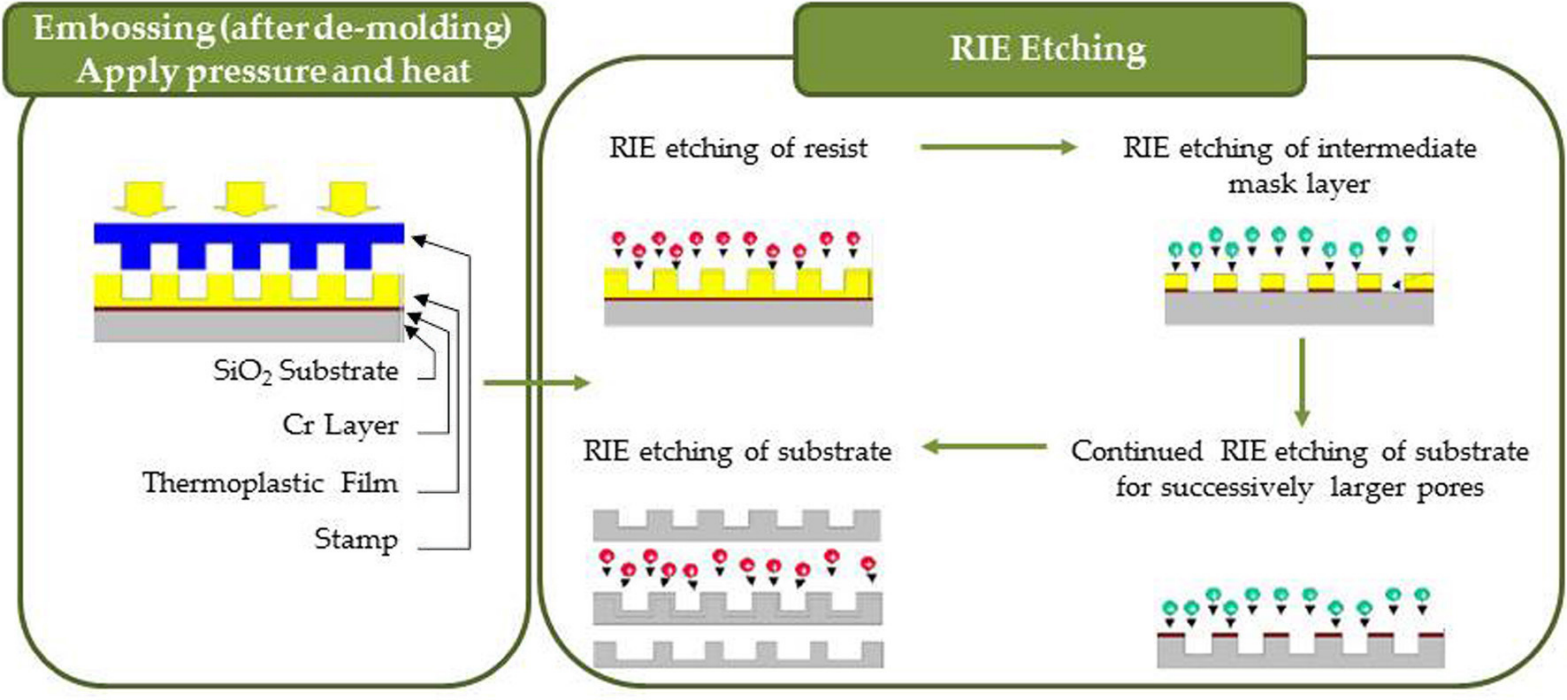
**Nanoimprinting process consisting of embossing and RIE etching into resist, intermediate mask layers and substrate**.

**Figure 7:**
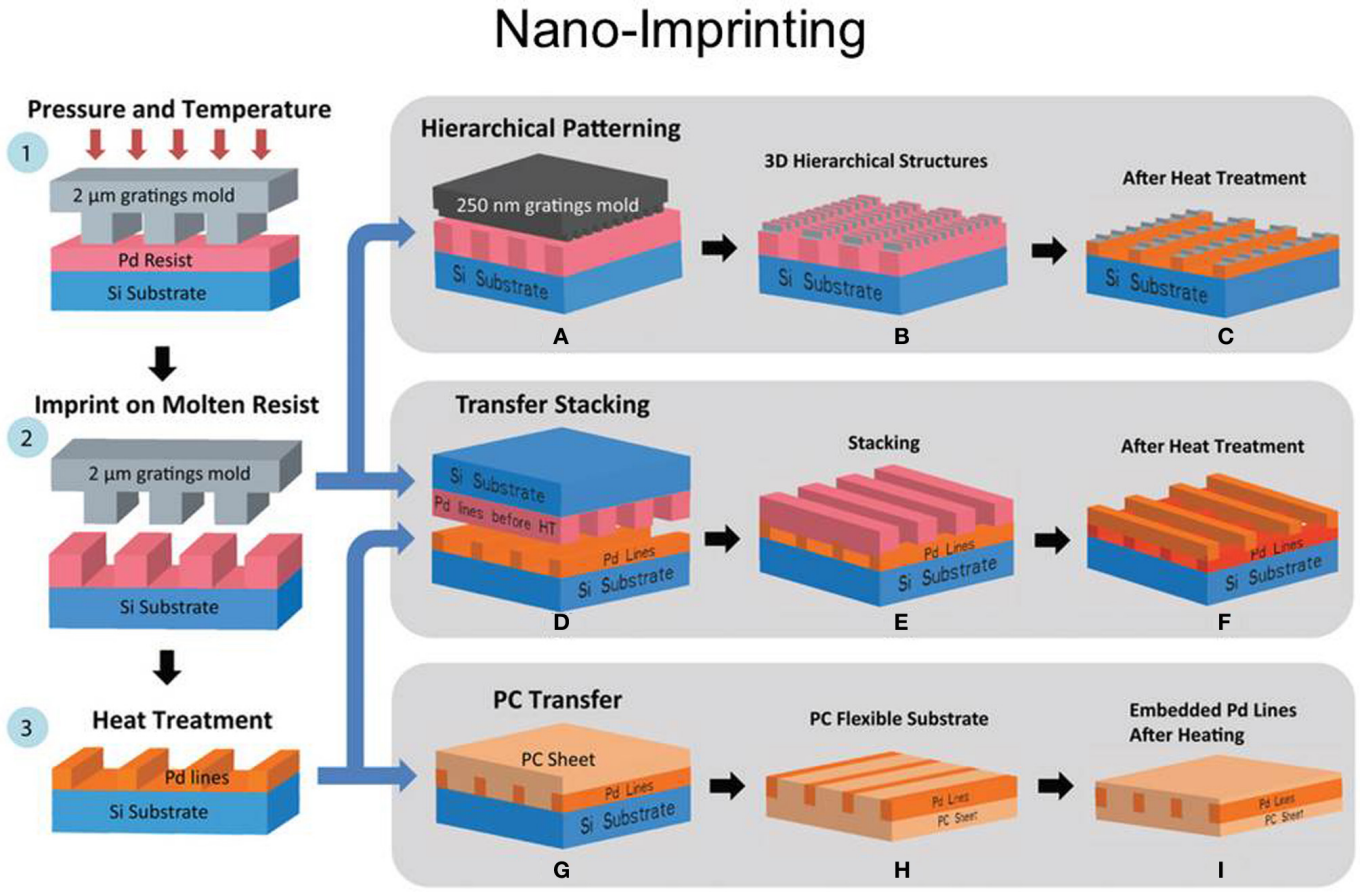
**Illustration of imprinting technique using a Si mold**. A Pd benzylthiolate film was spin coated on Si substrate and imprinted at temperatures close to its melting point (120řC) by applying pressure. During this process, the pattern of the mold is imprinted (2) and cooled down. After de-molding (3), the patterns on the Si substrate were heat treated to obtain the final Pd patterns. During stage 2, there are a few other patterning possibilities: (A–C) hierarchical patterning can be obtained by using a different Si mold with smaller feature sizes on top of the imprinted Pd benzylthiolate patterns; (D–F) transfer stacking that is realized by using the Pd pattern and (3) as a substrate; (G–I) polycarbonate (PC) transfer that is obtained by using (3) as mold and PC as substrate. (Reprinted by permission from Macmillan Publishers Ltd: Radha et al., 2012).

Recently, nanoimprint technology (NIL, Figure 6), has reproduced nanopatterns on large areas at a much lower cost than e-beam lithography by using mechanical embossing of a polymer at processing temperatures above glass transition temperature. Moreover, NIL technique can resolve patterns beyond light diffraction or beamscattering limitations as in other lithography techniques (Figure 7, Radha et al., 2012). Since it was demonstrated that NIL can achieve a sub-10 nm resolution and alignment (Chou and Krauss, 1997; Chou et al., 1997) with high fidelity on a large area pattern (Khang and Lee, 1999; Perret et al., 2004), this imprinting technique has been applied to produce microfluidics and microelectromechanical system (MEMS) devices, compact disks, field effect transistors, patterned magnetic disks, micro-optics, etc. Additionally, using NIL to create templates offers the benefit of creating templates of desired geometry for further optimization if needed.

Using NIL to create nanoelectrodes allows control of the nanostructure size (height, diameter), density, distribution and integration (Kuo et al., 2003; Torres et al., 2003; Hu et al., 2005; Konijn et al., 2005; Tormen et al., 2005; Cui and Veres, 2006; Le et al., 2006; Nakamatsu et al., 2006; Park et al., 2006; Sandison and Cooper, 2006; Zhang et al., 2006). Nanostructure fabrication is flexible in terms of choices of deposition techniques and the substrates on which the deposition takes place (insulators, semiconductors or conductors) and standard thin film fabrication techniques can be used. There is a need for new device architecture that requires less power and uses smaller surface than the multi-channel devices produced by lithographic patterning. Recently, Rehman and Kamboh (2013) reported a novel architecture to amplify the neural signal in implantable brain machine interfaces, which is able to manage both components of the neural signals, i.e., the action potentials, also known as neural spikes, and the local field potentials. Performance metrics could be improved if nanotechology is used to create novel architectures with nanosize features.

Although the template synthesis has been successfully used to create nanostructures such as nanorods and nanocable structures (see Figure 5), the integration of multiple junctions in a nanocable format is more challenging. Because this technology works for more simple nanostructures, the challenge is to finely tune the deposition conditions for creating multi-junction nanostructures using a diameter-controlled synthesis inside the nanopores of a custom made template. The unique properties of these metal core/multi-shell nanocable heterostructures combined with the possibility of building 3D interface between electrodes and neural tissue has great potential for applications at smaller scale than was previously possible.

**Figure 8:**
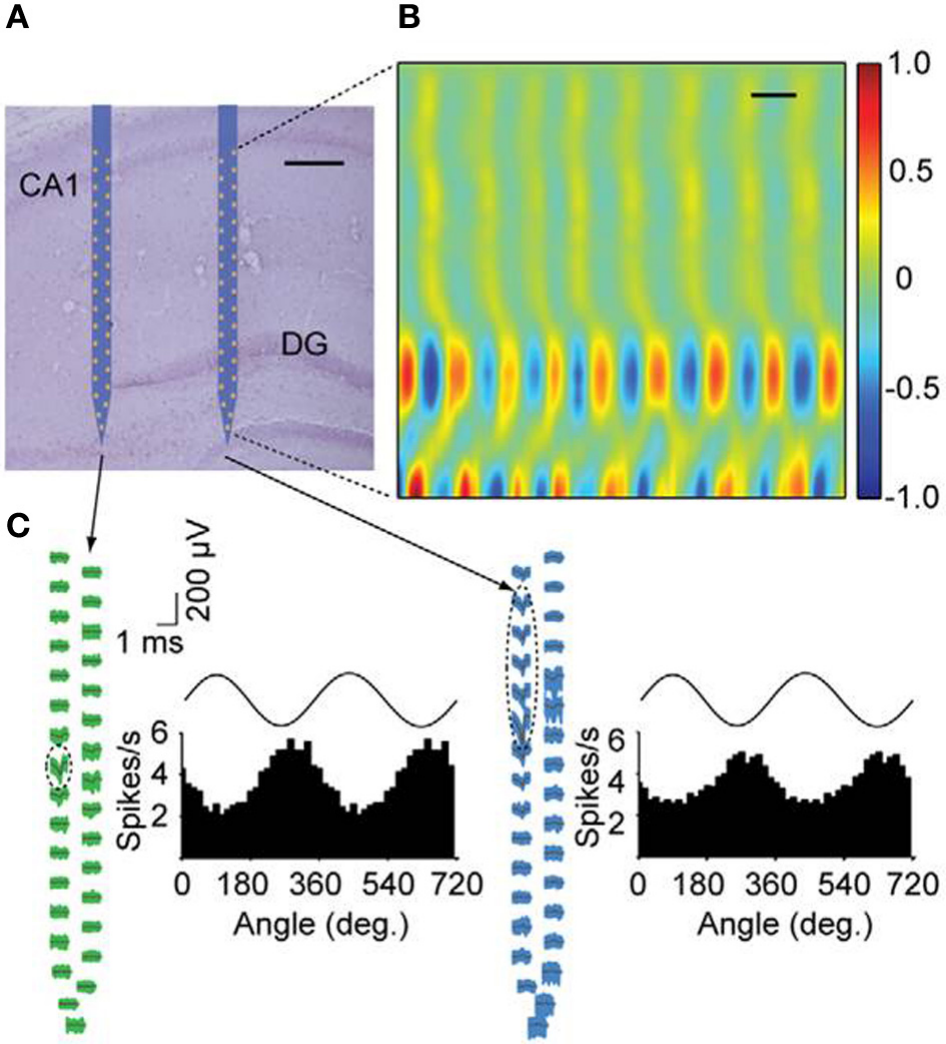
**Simultaneous recordings of neurons with nanofabricated probes in awake behaving mice. (A)** Nissl-stained brain section overlaid with a schematic of the probe at its stereotaxically implanted location. Each silicon shaft is 60 mm wide. Scale bar, 200 mm. **(B)** Current source density analysis of local field potentials across the hippocampus with a vertical resolution of 28 mm. DG, dentate gyrus. Neural data was collected during home cage exploratory behavior. Current source density is normalized to ś 1. Scale bar, 100 ms. **(C)** Waveforms of two putative single cells recorded with the nano-probe across all sites together with histograms showing theta phase locking of spikes. Dashed ellipses indicate the sites exhibiting the highest extracellular action potentials for these units. Theta oscillations shown on top of the histograms are for reference. Theta oscillations were measured from the upper rightmost electrode near the CA1 pyramidal cell layer (Adapted with permission from Du et al., 2011).

An example is the nanofabrication of extracellular electrode array with high density electrical leads such as the low noise multi-channel silicone system (Du et al., 2011) presented in Figure 8. These results were obtained in awake, behaving, mice by using nanoarrays with a 64 channel silicone-based neural probe. In the quest to minimize the size and the noise of the system, researchers are searching to decrease the size of neural probes. NIL could be a useful tool to increase the density of recording channels and to achieve high performance recording devices at small scale.

Engineering a nanostructure-integrated neuroelectrode represents a particularly difficult challenge: characterization by conventional methods reveals the complexity of materials while in fact the devices are quite simple. Unconventional physical properties are expected since the crystalline structure depends on the direction of growth. Device performance cannot be exclusively defined by taking the properties of each component and then applying junction theory to them. Instead, a multidisciplinary approach is required. Given the ability of nanoelectrodes to interact with physiological systems, there is a still work to be done in understanding the relationship between the nanostructure properties and the device properties made from them. Cooperative efforts of many techniques and approaches are required to gain a comprehensive understanding of this relationship. Additionally, many surface-sensitive techniques have been developed for flat and ultraflat surfaces but not for nanostructures.

## Nanostructures: a platform for brain repair and augmentation

The tools needed to study the brain must operate at the same nanoscale as brain functions. Nanoscience together with nanotechnology bring together a “rich toolkit” of unique methods useful for realizing the complexity of brain functions by allowing concurrent recording of thousands of neurons with manipulation of the activity of millions of cells. Huge effort is now devoted to the decoding of specific neural interactions and circuits, a goal that has emerged as the Brain Activity Mapping Project (Alivisatos et al., 2013). Several examples of the synergy between nanoscience and neuroscience contributions to brain study and brain augmentation are given as follows:

### Multi-electrode array (MEA) technologies for neuroscience

Using “substrate-integrated” microelectrode arrays (MEAs) is the finest approach to study brain circuitry, connectivity, neurophysiology, or pathology both *in vivo* and *in vitro*. MEAs add versatility to the real-time, long-term recording of chemical fluctuations in the extra-cellular micro-environment along with neurophysiological activity while being minimally invasive (Wise, 2007; Chang-Hsiao et al., 2010; Amaral et al., 2013). The organization of neural network, its neuronal excitability, and synaptic plasticity, together with drug responses may be monitored by MEAs.

#### Carbon nanotubes MEAs

Carbon nanotubes (CNTs) array chips are now used for non-invasive measurement of action potentials, real-time concentration of dopamine, and postsynaptic potentials. Suzuki et al. (Suzuki et al., 2013) developed MEA chips of planar CNTs that can measure both the electrophysiological responses (such as action potentials and field postsynaptic potentials) and release of the dopamine neurotransmitter. These CNTs-MEA chips have been fabricated directly on the microelectrode surfaces by electroplating an indium-tin oxide. Chronoamperometric measurements based on such CNTs-MEA chips detected dopamine concentration at nanomolar level with high temporal resolution and a 100-fold better signal to noise ratio. MEA chips may be useful for various applications such as drug screening and toxicity, *in vitro* stem cell differentiation, synaptic plasticity, or pathogenic processes associated with stroke, epilepsy, Alzheimer's and other neurodegenerative diseases.

#### Multi-walled carbon nanotubes MEAs

MEAs using MWCNTs have the advantage of decreased physical size of microelectrode with increased impedance and decreased charge-transfer capability (Gacem et al., 2013). To decrease impedance, the effective surface area for recording of the electrode needs to be increased. With a steam-plasma treatment the surface of MWCNTs becomes converted from super-hydrophobic to super-hydrophilic. This hydrophilic property is attributed to the OH bonding on the surface of MWCNTs. A multi-walled MEA was employed to record neural signals from a lateral giant cell of an American crayfish. This electrode type allows the separation of neural signals with their distinct shapes for long-term recordings and improved recording performance.

#### Multiplexed high density MEA

Recent neural probes based on silicon (Du et al., 2011) employed nanofabricated, high-density electrical leads that can read out multichannel data. MEA uses an application-specific integrated circuit (ASIC) to intensify signals, multiplexing functions and band-pass filtering. Multiplex high density devices with a fully integrated low noise, 64-channel system can perform high spatial resolution extracellular measurements and weighs just 330 mg (Du et al., 2011). These on-chip multiplexers allow recordings with “substantially fewer external wires than the number of input channels.” The combination of ASICs and nanofabricated probes that is both “minimally invasive and highly scalable” (Du et al., 2011) was employed for carrying out large-scale, high-density electrophysiology in small animals.

Similarly, Viventi et al. (2011) integrated “ultrathin and flexible silicon nanomembrane transistors” into a MEA, enabling “dense arrays” of thousands of amplified and multiplexed sensors to be connected with fewer wires. This system was employed to record in cat the spatial properties of brain activity *in vivo*, including patterns of activity like sleep spindles, single-trial visual evoked responses or electrographic seizures. These developments might provide diagnostic and therapeutic brain-machine interface devices.

#### Nanowire-based electrode for acute neural recordings in the brain

A new kind of electrode is based on “structurally controlled nanowires,” for neurophysiological measurements *in vivo* (Xie et al., 2010). This electrode has a sensing part made of a thin metal layer deposited on epitaxial grown GaP nanowires. Suyatin et al. (Xie et al., 2010) realized the first functional NWs-based electrode. The team also has successfully tested the electrode by *in vivo* recordings in the cortex of rat in multiple brain implantations. This kind of electrode with a controllable geometry of nanowires now can be further used for the investigation of many *in vivo* functional properties in nanostructured neuronal interfaces.

#### Substrate-integrated microelectrode arrays

Current methodologies permit the simultaneous, long-term non-invasive recordings of extracellular field potentials, but not the sub-threshold synaptic potentials that are generated by single cells. Because intracellular recordings of the electrophysiological properties (sub-threshold action potentials, synaptic potentials and membrane oscillations) can be acquired only by sharp (or patch) microelectrodes, these recordings may be limited to very short durations and single cells at a time. An emerging approach in a number of laboratories is based on the merging of the advantages of extracellular microelectrode arrays with those of intracellular microelectrodes (Spira and Hai, 2013).

### Brain-machine interfaces and neuroprosthetics

The neural prostheses that successfully help patients increase their daily living activities are quite simple implants that yield some definite tissue response and are well recognized as foreign body (Stieglitz, 2007). Based on the latest developments in materials science, new avenues for highly advanced systems to interface the human brain have emerged. Nanotechnology is opening the door to employing macromolecular approaches to implants that mimic the “biologic topology” and take into account the surface interaction of biologic cells. Combinations of neural cells with micro-implants can become the platform of stable bio-hybrid interfaces. Furthermore, converging technologies that exploit synergies between computer sciences and engineering, neuroscience and psychology are envisioned to completely change the understanding of the entire field.

Artificial synapses in neuromorphic circuits based on nanoscale memory devices have been recently accepted as a promising route for creating novel circuit architectures that tolerate variability and/or defects (Gacem et al., 2013). Still, the implementation of the neural network type of circuits that are based on non-CMOS (complementary metal-oxide-semiconductor) memory devices with learning capabilities are rare. Gacem et al. (2013) showed that memory elements based on CNTs may be used as “artificial synapses” combined with “conventional neurons” further “trained” to perform several functions (by applying a supervised learning algorithm). This is possible because the same device ensemble can be trained many times to code successively any type of 3-input combination of Boolean logic functions despite the variability among devices. This approach has huge potential for application to parallel learning of several devices with more complex function.

Carbon nanowires used as interface material in contact with neurons can both deliver electrical stimulation to these cells and detect neuronal electrical activity. Carbon nanowires or nanotubes emerge as materials that do not have recognizable adverse effects. Consequently, they can be successfully used in brain-machine interfaces (Malarkey and Parpura, 2010). In recent years, research on growing CNT substrates has been used to examine *in vivo* formation of neurons and neuronal networks during guided growth by artificial nano-scaled cues. Additionally, prostheses for monitoring brain activity were developed using interfaces based on nanotube architecture (Stankova et al., 2014). Fabbro et al. (2012) demonstrating the alteration of various hippocampal neurons responses by the CNT substrates in cultures. This observation highlighted the exceptional ability of the CNT substrate to induce nerve tissue growth. They confirmed that CNT scaffolds promote the development of immature neurons isolated from the neonatal rat spinal cord and maintained *in vitro*. by performing electrophysiological studies associated with gene expression analysis. Results indicated that spinal neurons plated on electro-conductive CNTs show an assisted expansion. These microarray experiments suggest that CNT platforms activate healing activities involving microglia in the absence of reactive gliosis.

### Application of carbon nanotubes imaging

Imaging applications of CNTs to living cells and tissues bring promising advantages to biological applications based on optical properties of nanotubes. Due to the high photostability of SWCNTs' photoluminescence, a longer excitation time is attainable at higher laser fluency compared to quantum dots or organic fluorophores. Also, attenuated absorption combined with autofluorescence and scattering characteristics makes visible the opaque tissue in the range of 700–1400 nm, while nanotubes allow imaging of the whole blood and thick tissue (Heller et al., 2006). This is mainly because the fluorescence profiles of many semiconducting NTs overlaps with the wavelength range. Imaging SWCNTs in tissue sections and the nanotubes concentration measured in blood was also based on nanotube fluorescence (Cherukuri et al., 2006). In addition, CNTs can be detected due to their large resonance-enhanced Raman scattering (Heller et al., 2005).

### Inter-laminar microcircuits across neocortex: repair and augmentation

**Figure 9:**
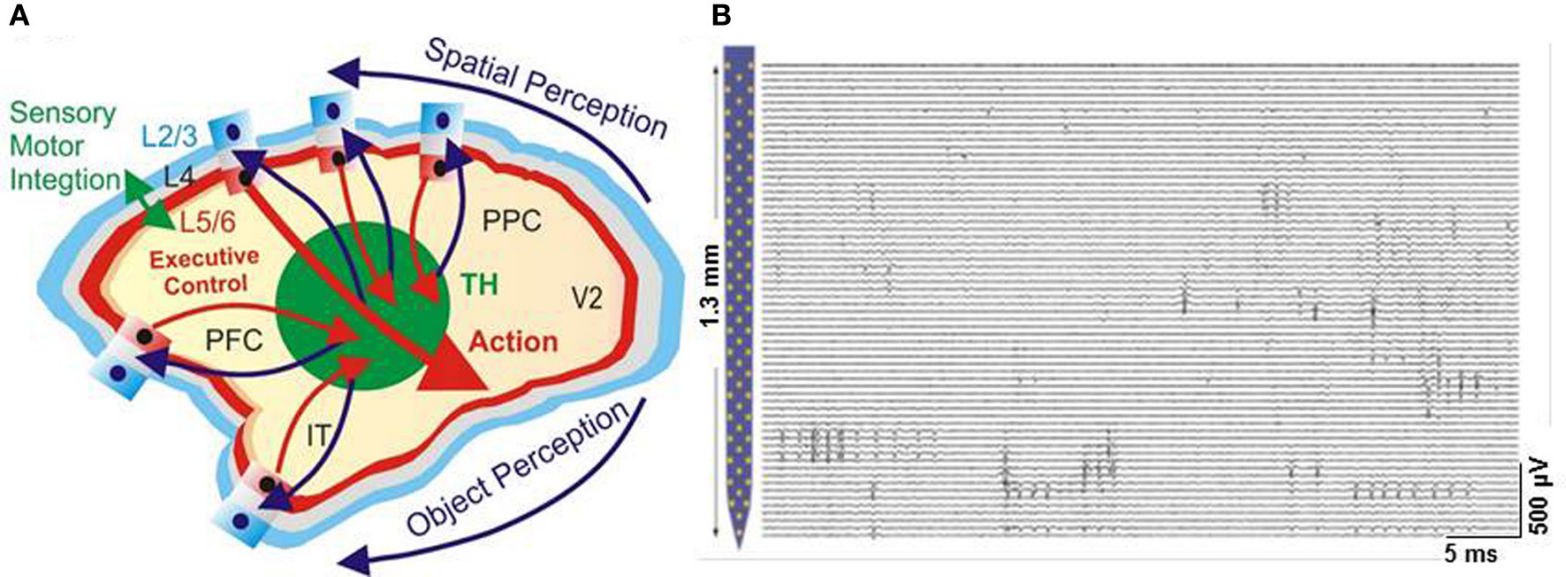
**Cortical modularity and parallel recording capabilities of the multiplexed neurophysiological system. (A)** Cortical modularity showing laminar anc columnar arrangements of neurons in the primate neocortex (L2/3 is layer 2/3, L5/6, layer 5/6, V2, visual cortical area 2, PPC, posterior parietal cortex, PFC, prefrontal cortex, IT, inferotemporal cortex, TH, thalamus). **(B)** Parallel recording with nano-array in the mouse brain (Adapted with permission from Du et al., 2011).

Repair and brain augmentation approaches, such as brain-machine interfaces (Nicolelis et al., 2003; Lebedev and Nicolelis, 2006; Opris, 2013), neural stimulation and other neural prostheses, have experienced rapid development during the last decade (Opris and Bruce, 2005; Lebedev and Nicolelis, 2006; Opris et al., 2013). However, few of these methods target the inter-laminar micro-circuitry of the brain (Jones and Rakic, 2010; Opris et al., 2011, 2012a,b). The potential for employing inter-laminar recording and micro-stimulation of cortical microcircuits with CNT-MEAs to build neural prostheses for repair and augmentation of cognitive function is now being considered. Thus, nanotechnology is instrumental to nanofabricate planar electrode arrays (Figure 9) to be used in high-density neuronal voltage recording (Du et al., 2011; Suyatin et al., 2013). Micro/nano-fabrication technologies raise the prospect for increasing the numbers of electrodes for smaller, less invasive implantable devices. A promising nano-array for brain microcircuits is the new planar electrode array (Viventi et al., 2011; Alivisatos et al., 2013), which is configured on a crystalline, ceramic, or polymer support structure (Figure 9). Recording neural firing with 3-dimensional microelectrode arrays (Zorzos et al., 2012) represents a major advance in brain activity mapping techniques, by providing a tool to demonstrate how intra and inter-laminar/regional neural circuits cooperate together to process information. Building prosthetic minicolumns (Mountcastle, 1957, 1997; Buxhoeveden and Casanova, 2002; Mahan and Georgopoulos, 2013; Opris and Casanova, 2014) as basic modules to repair the damaged cortical tissue will become a valuable approach for cognitive neuroprosthetics. This may be accomplished by designing artificial minicolumns that can be inserted by minor surgery into the human brain, or the use of nanowire contacts to place a device with minicolumn function within the damaged circuitry (Lebedev and Nicolelis, 2006; Bokara et al., 2013; Marmarelis et al., 2014). Moreover, neural enhancement approaches may be applied to inter-laminar microcircuits across the entire cortex (Opris, 2013). In the future, such microcircuit based prostheses will provide efficient therapies for patients with neurological and psychiatric disorders (Casanova, 2007, 2013; Casanova et al., 2008; Chance et al., 2011).

### Associative memories with enhanced storage capacity

The modeling of associative memories with non-monotonic neural networks (Nishimori and Opris, 1993) was demonstrated by Monte Carlo computer simulations to yield an enhanced storage capacity. Recent experiments employing MIMO micro-stimulation (based on multiplexing principle) of prefrontal cortical inter-laminar microcircuits was shown to enhance cognitive performance and memory in non-human primates performing a behavioral task (Hampson et al., 2012; Opris et al., 2012a,b). Future use of multiplexed high density MEAs (Du et al., 2011) holds the promise to provide an unprecedented enhancement of memory for both artificial intelligence and humans with implanted chips.

### Biocompatibility between carbon nanotubes and stem cells

Regenerative medicine, especially for CNS, has looked extensively into the possibility to use stem cell therapy to replace lost cells during CNS injuries (Bokara et al., 2013). However, the survival rate of the transplanted stem cells affecting tissue restoration may be limited by the toxic byproducts and the complexity of the CNS injuries. CNTs are a novel class of nanomaterials that show encouraging results in various areas of nanomedicine including therapy, diagnosis, and prevention of CNS diseases. The use of CNTs as substrates or scaffolds in the study of stem cell differentiation has recently become a dynamic research area. Both SWCNTs and MWCNTs are being used more often as scaffolds for neural stem cell differentiation and for neuronal growth. The use of CNTs-based materials was shown to affect the differentiation of progenitor and stem cells and to guide them in the direction of specific neurons. In addition, they enhanced synaptogenesis and axon regeneration, while being effective in the treatment of brain injuries. Moreover, increasing evidence supports the great potential of CNTs in neurobiological research and their use as a tolerant and biocompatible substrate/scaffold for neural cells.

### Nanotechnologies used for stem cells

Nanotechnologies have emerged as useful platforms for understanding, measuring, and manipulating stem cells (Ferreira et al., 2008). Examples include: (i) magnetic nanoparticles (NPs) and quantum dots for labeling stem cell and *in vivo* tracking, (ii) NPs, CNTs, and polyplexes used for the intracellular delivery of genes (i.e., oligonucleotides) and protein (i.e., peptides), (iii) nanometer-scale engineered scaffolds for stem cell differentiation and transplantation, and (iv) nanotechnological use for tracking, differentiation, and transplantation of stem cells.

## Conclusion

Nanostructures are platforms that can be used in many ways for brain repair and augmentation. In addition, these nanostructures play a crucial role in the study of brain disorders. One exciting approach is to integrate them into arrays for developing sensors and biomarkers. Due to the unique electrical and optical properties of nanowires, nanotubes, and nanocables assembled on sensing platforms, they also hold the potential to augment brain functions. There are many challenges in creating nanowires/nanotubes/nanocables arrays-based sensors, but the goal is to make individual electrical connections between brain microcircuits and nanostructures of interest. Finally, nanostructures represent the interface between nanotechnology and neuroscience, making them promising aids in neurology for the diagnosis and treatment of brain disorders.

### Conflict of interest statement

The Reviewer Dr Song declares that despite having collaborated with the author, Dr Opris, the review process was handled objectively. The authors declare that the research was conducted in the absence of any commercial or financial relationships that could be construed as a potential conflict of interest.
